# Impact of Ultrasonography on Chest Compression Fraction and Survival in Patients with Out-of-hospital Cardiac Arrest

**DOI:** 10.5811/westjem.2023.1.58796

**Published:** 2023-02-27

**Authors:** Wan-Ching Lien, Kah-Meng Chong, Chih-Heng Chang, Su-Fen Cheng, Wei-Tien Chang, Matthew Hwei-Ming Ma, Wen-Jone Chen

**Affiliations:** * National Taiwan University Hospital, Department of Emergency Medicine, Taipei City, Taiwan, Republic of China; † National Taiwan University, College of Medicine, Department of Emergency Medicine, Taipei City, Taiwan, Republic of China

## Abstract

**Introduction:**

Whether ultrasonography (US) contributes to delays in chest compressions and hence a negative impact on survival is uncertain. In this study we aimed to investigate the impact of US on chest compression fraction (CCF) and patient survival.

**Methods:**

We retrospectively analyzed video recordings of the resuscitation process in a convenience sample of adult patients with non-traumatic, out-of-hospital cardiac arrest. Patients receiving US once or more during resuscitation were categorized as the US group, while the patients who did not receive US were categorized as the non-US group. The primary outcome was CCF, and the secondary outcomes were the rates of return of spontaneous circulation (ROSC), survival to admission and discharge, and survival to discharge with a favorable neurological outcome between the two groups. We also evaluated the individual pause duration and the percentage of prolonged pauses associated with US.

**Results:**

A total of 236 patients with 3,386 pauses were included. Of these patients, 190 received US and 284 pauses were related to US. Longer resuscitation duration was observed in the US group (median, 30.3 vs 9.7 minutes, P<.001). The US group had comparable CCF (93.0% vs 94.3%, P=0.29) with the non-US group. Although the non-US group had a better rate of ROSC (36% vs 52%, P=0.04), the rates of survival to admission (36% vs 48%, P=0.13), survival to discharge (11% vs 15%, P=0.37), and survival with favorable neurological outcome (5% vs 9%, P=0.23) did not differ between the two groups. The pause duration of pulse checks with US was longer than pulse checks alone (median, 8 vs 6 seconds, P=0.02). The percentage of prolonged pauses was similar between the two groups (16% vs 14%, P=0.49).

**Conclusion:**

When compared to the non-ultrasound group, patients receiving US had comparable chest compression fractions and rates of survival to admission and discharge, and survival to discharge with a favorable neurological outcome. The individual pause was lengthened related to US. However, patients without US had a shorter resuscitation duration and a better rate of ROSC. The trend toward poorer results in the US group was possibly due to confounding variables and nonprobability sampling. It should be better investigated in further randomized studies.

## INTRODUCTION

Chest compressions, the most important maneuver during cardiopulmonary resuscitation (CPR), generate cardiac output and maintain vital organ perfusion.^1^ Interruption of chest compressions impairs coronary and cerebral perfusion and compromises the outcome of resuscitation.^2^,^3^ High quality CPR with minimized interruptions is a cornerstone of successful resuscitation for patients with cardiac arrest (CA). Current resuscitation guidelines recommend that a single pause for a pulse check should not exceed 10 seconds.^4^

Chest compression fraction (CCF), an index indicator for the quality of CPR, is defined as the proportion of the time spent providing chest compressions during the whole CPR process. A positive benefit from CCF on the rate of return of spontaneous circulation (ROSC) was reported, although the ceiling effect of CCF was at 80%.^4^–^6^ A variety of actions or procedures were related to interruptions of chest compressions, such as pulse checks, defibrillation, intubation, change of personnel performing the compressions, application of CPR adjuncts, etc.^1^,^7^,^8^

Ultrasonography (US), given its characteristics of non-invasiveness and accessibility, exhibits value in critical conditions such as CA and shock.^9^–^12^ Current guidelines for Advanced Cardiovascular Life Support (ACLS) suggest that US can be an integral part of the resuscitation process.^13^ Despite its potential in identifying reversible causes, concerns arise regarding whether US contributes to delays in chest compressions and hence a possible negative impact on patient survival. Previous studies have shown that US prolonged a single pause to 21 seconds, ranging from 13–24 seconds,^14^,^15^ although the pause could be shortened if the US was performed by a well-trained sonographer.^15^,^16^ The benefit contributing to US and the risk of chest compression delays could be balanced.

We conducted a study to investigate the impact of US on CCF and patient survival among patients with out-of-hospital cardiac arrest (OHCA). We also evaluated the individual pause duration and the percentage of prolonged pauses associated with US.

## MATERIALS AND METHODS

### Study Design and Setting

This retrospective study was conducted from April 2017–March 2019 in the emergency department (ED) of National Taiwan University Hospital The protocol was approved by the Institutional Review Board of the hospital’s Ethics Committee with a waiver of informed consent, and the study was registered at ClinicalTrials.gov (NCT03695536).

Patients with OHCA were directly transported by emergency medical services to the resuscitation rooms. An organized team was responsible for the resuscitation of the patients at a designated resuscitation area. The resuscitation team was composed of two senior emergency physicians (an attending physician/senior resident as the team leader, with the other for the airway), two junior emergency residents (responsible for chest compressions or defibrillation), and four senior nurses (one for management of airway and ventilation, one for vascular access, one for drug preparation, and one for recordings of the CPR process). All ACLS-certified personnel had pre-allocated roles and tasks.^17^ All resuscitation was performed according to the ACLS guidelines.^18^

**Population Health Research Capsule**

What do we already know about this issue?

*Whether ultrasonography (US) contributes to the delays in chest compressions and hence a negative impact on survival is uncertain*.

What was the research question?

*What is the impact of US on chest compression fraction (CCF) and patient survival?*

What was the major finding of the study?

*The US cardiac arrest group had comparable CCF (93.0% vs 94.3%, P=0.29) with the non-US group. Rates of survival to admission (36% vs. 48%, P=0.13) and to discharge (11% v. 15%, P=0.37) were also similar*.

How does this improve population health?

*Patients receiving US had comparable CCFs and rates of survival to discharge with a favorable neurological outcome with the non-US group*.

Overhead video cameras in the resuscitation room had previously been approved to record the CPR process for regular quality review and assurance for more than 10 years. The video recordings were stored in a secured hospital database. Also, a timer was routinely employed during CPR with a regular alarm every two minutes as a reminder to check pulse, and 10 seconds thereafter for resumption of chest compressions. A Noblus US machine (Hitachi Aloka Medical, Ltd, Tokyo, Japan) equipped with 2–5 megahertz curvilinear transducers was kept ready for use in the resuscitation room.

The senior residents who completed basic emergency US training (certified by the Taiwan Society of Emergency Medicine, Supplementary file) and resuscitative US training (the US-Compression Airway Breathing (CAB) training curriculum, Supplementary file)^19^ performed sonographic examinations during CPR. All of them had passed the immediate evaluation and the re-evaluation six months later in the simulation settings. They also passed the evaluation in real resuscitation settings and showed their competency in our previous work.^19^,^20^ The cardiac US was routinely performed to detect sonographic cardiac activity after 10 minutes of CPR.^20^

### Patient Inclusion

Adult patients >20 years of age (per the Regulations on Human Trials conducted since 2016 in Taiwan) with non-traumatic OHCA were eligible for inclusion. A convenience sample of patients receiving US during resuscitation was included when trained sonographers were available. Patients not receiving US were included in the same month. Exclusion criteria were patients <20 years old, traumatic CA, and do-not-resuscitate (DNR) orders.

### Data Collection

The video recordings of resuscitation were downloaded to an encrypted hard drive for retrospective review, and the faces of the resuscitation team members were masked. Each pause, including pause duration and associated activities, was analyzed and recorded by two emergency physicians who were blinded to the study hypothesis, not involved in resuscitation and ultrasound training, and had more than 10 years ED practice. If disagreement occurred, a third member was consulted until consensus was achieved. We recorded the total time spent on chest compressions and in-hospital resuscitation duration from the start of video recording to the end of resuscitation.

The CCF was defined as the fraction of time spent on chest compressions during the in-hospital CPR process. The rate of chest compressions was measured using a timer together with a counter. The percentage of prolonged pauses was defined as the percentage of pause durations of more than 10 seconds.

The clinical information of the patients, including age, gender, past medical history, witness status on CA, bystander CPR, prehospital CPR duration, initial cardiac rhythm, the cause of CA, the doses of epinephrine, and patient survival were obtained from the electronic health records. The cause of CA included cardiovascular (myocardial infarction, pericardial effusion, abdominal aortic aneurysms, dissecting aortic aneurysm, etc); airway (sputum impaction, aspiration, pneumonia, etc); sepsis; and others (malignancy, hyperkalemia, hypotension, hypoglycemia, gastrointestinal bleeding, etc). A favorable neurological outcome was defined as a Glasgow-Pittsburgh Cerebral Performance Category score of 1–2. The emergency physicians who were blinded to the study hypothesis, not involved in resuscitation and ultrasound training, and had more than 10 years of ED experience, reviewed the medical records.

### Outcome Measurement

Patients receiving US once or more during resuscitation were categorized as the US group and those not receiving US were categorized as the non-US group. The primary outcome was CCF and the secondary outcomes were the rates of ROSC, survival to hospital admission, and survival to hospital discharge between the two groups. We also assessed the individual pause duration and the percentage of prolonged pauses associated with US.

### Sample Size Estimation

We used SAS analytics software version 9.4 (SAS Institute, Inc, Cary, NC) for sample size calculation. We assumed the proportion of patients receiving US during resuscitation was 67.6%.^21^ With a power of 0.8 and a 5% significance level, the calculated sample size was 30 patients for each group.

### Statistical Analysis

We analyzed all data using SAS. Initially, we used the Shapiro-Wilk test for the normality of continuous data. If the data was not normally distributed, it was expressed in medians and interquartile (IQR) ranges and examined using Wilcoxon’s rank-sum test. Categorical data was expressed in counts and proportions and compared using a chi-square test or Fisher’s exact test. Intraclass correlation (ICC) with 95% confidence intervals (CI) was used to assess interrater reliability for each pause by two physicians.

To investigate the possible factors associated with the patients receiving US, we further incorporated the factors of statistical significance in univariate analysis in multiple logistic regression analyses. The covariates in the regression model included age, gender, witnessed arrest, bystander CPR, prehospital CPR duration, defibrillation during Basic Life Support (BLS) and ACLS, cardiovascular etiology, doses of epinephrine, and in-hospital resuscitation duration.

Additionally, to investigate the possible factors associated with patient outcomes including ROSC, survival to admission, and survival to discharge, we further incorporated the factors of statistical significance in univariate analysis in multiple regression analysis. The covariates in the regression models included age, gender, witnessed arrest, bystander CPR, prehospital CPR duration, defibrillation during BLS and ACLS, intubation, cardiovascular etiology, doses of epinephrine, and in-hospital resuscitation duration, and the use of US. We computed odds ratios (OR) with 95% CIs.

Since there were numerous pauses on each patient during resuscitation, a within-subject correlation on pause length would exist. We applied repeated measures using a mixed model to compare the pause durations associated with US in the US group. Covariates in the mixed models included group (with or without US), and the number of times associated with certain activities. A *P*-value of less than 0.05 was considered statistically significant.

## RESULTS

### Characteristics of Study Subjects

We collected data on 320 adult patients with OHCA from April 2017–March 2019. After excluding the patients with trauma and DNR orders, we included 236 patients in the current analysis (Figure). Ninety-two patients (39%) achieved ROSC, 90 patients (38%) survived to admission, 27 (11%) survived to discharge (Figure) and 13 (6%) survived with favorable neurological outcomes. A total of 190 patients received US once or more during resuscitation.

There was good interrater reliability in the pause duration with an ICC of 0.92 (95% CI 0.85–0.96) and the associated activities with an ICC of 0.86 (95% CI 0.80–0.95).

After the examination of normality, age, prehospital CPR duration, in-hospital resuscitation duration, in-hospital pauses, the dose of epinephrine, chest compression rate, and CCF (all *P*<0.0001) were not normally distributed and presented with medians and IQRs. See Table 1 for patient demographics. No significant differences were noted in age, gender, and underlying medical diseases between the two groups. The median timing of US was at the eighth minute of CPR (IQR, 6^th^–12^th^ minute).

A greater percentage of the arrests was attributed to cardiovascular etiology in patients receiving US (53% vs 28%, *P*<.001). Among them, dissecting aortic aneurysms, massive pericardial effusion, and ruptured abdominal aortic aneurysms were diagnosed in 12 patients with the aid of US. One patient with dissecting aortic aneurysm was sent to the operating room following ROSC, and five with pericardial effusion received pericardiocentesis. Also, sonographic cardiac activity was detected in 85 patients receiving US. Of 68 patients achieving ROSC, 64 had sonographic cardiac activity. Patients with sonographic cardiac activity exhibited a higher chance of ROSC (64/68 vs 21/122, *P*<0.001).

### Chest Compression Fraction

Ultrasound was not associated with a lower CCF, although longer in-hospital resuscitation duration was observed in the US group (Table 2). There was no significant difference in witnessed arrest, bystander CPR, prehospital CPR duration, initial shockable rhythm, defibrillation, intubation, the dose of epinephrine, and chest compression rate between the two groups.

The univariate regression analysis showed that cardiovascular etiology (OR 2.78, 95% CI 1.06–7.28) and longer resuscitation duration (OR 1.08, 95% CI 1.03–1.13) were associated with the use of US. Longer resuscitation duration (OR 1.08, 95% CI 1.03–1.13) remained significant after adjusting cardiovascular etiology in the multiple regression analysis (Table 3).

### Patient Outcomes

Although patients not receiving US had a better rate of ROSC (36% vs 52%, *P*=0.04), the rates of survival to admission (36% vs 48%, *P*=0.13), survival to discharge (11% vs 15%, *P*=0.37), and survival with favorable neurological outcome (5% vs 9%, *P*=0.23) did not differ between the two groups (Table 4). The significant factors associated with patient survival are shown in Supplementary Table 1. Longer in-hospital resuscitation duration was associated with less chance of ROSC, survival to admission, and survival to discharge after adjusting other parameters.

### Pause Duration and the Percentage of Prolonged Pauses Associated with Ultrasound

There were 3,386 pauses analyzed in this study. The details of activities during pauses are listed in Supplementary Table 2. Pulse checks were the most common activities. and all US was performed during pulse checks.

In the US group, US was performed in 284 (15%) of the 1,835 pulse checks. A mixed model was applied to clarify intra-patient correlation and time-dependent effects. Covariates included the use of US and the number of times for pulse checks. Among patients receiving US, the pause duration of pulse checks with US was longer than pulse checks alone (median, 8 vs 6 seconds, *P*=0.02, Table 5). No time-varying effect was identified (*P*=0.16). No difference existed in the pause duration of pulse checks alone between the two groups (*P*=0.21).

The percentage of prolonged pauses was similar between the pulse checks alone and those with US (14% vs 16%, *P*=0.316) among patients receiving US. The percentage of prolonged pauses was also similar compared to those during pulse checks with US in the US group with those during pulse checks alone in the non-US group (16% vs 14%, *P*=0.49).

Notably, all the US was performed not only during the pause for pulse checks but extended into the next chest compression phase. The sonographic examination was focused on the heart during the pulse checks (Supplementary Table 3). Once chest compression was resumed, the sonographer either continued US scanning of the heart or switched to screen other targets such as the abdominal aorta or to scan for any existence of intraperitoneal free fluid.

## DISCUSSION

In recent decades, US has become a frequently used imaging tool during resuscitation. Many resuscitative US protocols such as the Cardiac Arrest Ultrasound Exam,^22^ Focused Echocardiographic Evaluation in Life Support (FEEL),^23^ the Sequential Echographic Scanning Assessing Mechanism protocol,^24^ Cardiac Arrest Sonographic Assessment (CASA) protocol,^16^ US-CAB,^20^ and Sonography in Hypotension and Cardiac Arrest (SHoC)^25^ were developed to search for potentially reversible causes of CA. Ultrasonography has been reported to prolong the duration of pause and delay the resumption of chest compressions.^14^,^15^ However, the evidence regarding US on overall CCF was limited. In this study, patients receiving US had CCF comparable with those who did not receive US. Although patients without US had a better rate of ROSC, the rates of survival to admission, survival to discharge, and survival with a favorable neurological outcome did not differ between the two groups. Ultrasonography was related to lengthening individual pause duration; however, the percentage of prolonged pauses was similar between the two groups.

Avoiding unnecessary interruptions of chest compressions and reducing pause duration have been repeatedly emphasized in this era of high quality CPR. In recent years, an even more important indicator, CCF, has been identified as a key benchmark of the quality of CPR.^26^ Previous studies have shown that increased CCF results in a higher rate of ROSC,^6^ although a ceiling effect exists once the CCF is greater than 80%.^5^,^27^ To date, the studies regarding US during CPR mostly reported the individual pause duration but not the CCF during the whole resuscitation process.^14^,^15^ Although the individual pause could be lengthened with the employment of US,^14^,^15^ the overall impact on CCF is not clear yet. This study showed the CCF in patients receiving US was similar to those without, possibly because US was performed in about 15% of pulse checks. Although the individual pause was prolonged with US, the overall CCF was not influenced.

The overall CCF was as high as 93% in this study, which was higher than the recommendation.^28^ Such a high CCF could be explained by adequate manpower, structured ACLS teamwork, and the employment of a timer reminding the resumption of chest compressions. In the current study, at least eight members were involved in each resuscitation scenario. The work of each member was pre-assigned and well-orchestrated. Also, the timer played a key role in reminding the team members to keep the pause as short as possible, even when US was being performed. Without such reminders, the leader and the sonographer would tend to concentrate on their work at hand and overlook the elapsed time. Moreover, proper US training and a readily available US machine are important. All the sonographers in this study completed the basic US and resuscitative US training beforehand. Through continued practice and accumulation of experience, the sonographers exhibited excellent US performance,^19^ even during CPR.

Moreover, a high quality portable US machine properly equipped and located in a resuscitation room is essential. This helps speed up imaging acquisition and interpretation. Further, US was performed not only during the pause period but extended to the next cycle of chest compressions. In previous studies, the US was performed during the pause for pulse checks only. If the sonographer tried to finish or extend the US exploration, the pause duration could be prolonged. On the contrary, if the sonographer allowed the resumption of chest compressions while continuing US examination in the following cycle,^29^ the pause duration could become shorter. Allowing resumption of compressions while continuing US largely broadened the time window for US assessment during CPR. Although US examination during the chest compression phase is much more challenging, the sonographer could take the chance of trying to complete the views that were not finished during the pause period. If chest compressions made the subxiphoid view of the heart not feasible, the sonographer could switch to other views checking the abdomen, chest, or other sites. In the current study, the aorta and any presence of intraperitoneal free fluid were the most often checked targets during the chest compression phase, while the subxiphoid view of the heart was mostly done during the pause period. Altogether the factors above would help make CCF highly compliant with the ACLS guidelines and shorten pause duration related to US, compared to the results reported in previous studies.^14^,^15^

In this study, we also focused on the effect of US on patient-centered outcomes. Although patients receiving US had a lower rate of ROSC, the effect of US was not significant for patient outcomes in the regression analysis. Witness arrest was positively associated with ROSC; by contrast, in-hospital resuscitation duration had a negative association with ROSC, survival to admission, and survival to discharge. It is noteworthy that our results showed that longer resuscitation duration was associated with the use of US. A similar phenomenon was reported in the previous research.^30^ This was reasonable since the longer the resuscitation without achieving ROSC, the more likely US would be employed during CPR searching for potentially reversible causes. On the other hand, it implies that the employment of US started after the standard resuscitation efforts or equipment had already been applied. With the retrospective nature of this study and convenience sampling, any conclusion that the use of US either improved or diminished the effectiveness of CPR could not be drawn. Further randomized studies would be needed to answer the question.

Although the pause duration during pulse checks with US was still longer than pulse checks alone in our study, the median duration was less than 10 seconds. The results were concordant with those in the FEEL study.^9^ By contrast, the PUCA study of paramedic-led echo in life support showed a pause duration of 17 seconds with US in prehospital settings.^31^ Previous studies showed that US prolonged a single pause to 21 seconds.^14^,^15^ However, as shown in the CASA study, this could be shortened by the implementation of the US protocol and the presence of US-trained faculty.^16^

Given that sonographic cardiac activity could be a prognostic factor for ROSC, the possible etiology of arrests could be identified or ruled out with the use of US. In patients receiving US, dissecting aortic aneurysms, massive pericardial effusion, and ruptured abdominal aortic aneurysms were detected in 12 patients and ruled out in the 178 remaining patients. However, mortality resulting from aortic dissection or rupture remained very high in arrest patients.^32^ Another important observation was that the chance of identifying reversible causes by US during CPR (such as cardiac tamponade, pulmonary thromboembolism, hypovolemia, acute coronary syndrome, et.) was generally low, making the chance of dramatic improvement by specific interventions much lower.

## LIMITATIONS

There were limitations in this study. First, the data was collected from a convenience sample and retrospective reviews. Missing data or abstractor bias could have occurred.^33^ There is a significant likelihood of selection bias, particularly regarding the imbalance in causes of CA between the groups. However, the results showed longer resuscitation duration was the only significant factor associated with the use of US after adjusting the confounders in the multivariate regression model. It implied the physician used US in a higher percentage of patients with sustained arrest to search for potentially reversible etiology after standard resuscitation efforts, reflecting the real scenario. Also, the faces of the resuscitation team members were masked and blinded to the researchers. The interrater reliability was fair. The chart abstractors were blinded to the study hypothesis. Nevertheless, a nonprobability sample would limit the interpretation of the findings. Further randomized trials would be needed to prevent certain bias.

Second, the study was conducted in a single center with well-structured ACLS teamwork and active US training. Notably, a timer was used in the resuscitation scenarios, reminding the physicians to avoid prolonged pauses. While as a whole the resources, assignments, and training of the clinicians demonstrated a high CCF, any extrapolation of the results would be uncertain. However, we provided a possible solution to lessen or avoid interruptions of CPR with the use of US. Future studies would be needed to test whether these results could be extrapolated to other settings.

Third, there were cameras in the resuscitation room. This would have introduced selection bias if patients received resuscitation outside that room. In this study, all the patients were resuscitated in the resuscitation room. There was the possibility of the Hawthorne effect due to the presence of cameras, although they had been in place for more than 10 years for quality control of CPR in our department. Fourth, the chest compression depth was not measured for CPR quality and could not be adequately interpreted using video review in the current study. This could be improved by incorporating optical sensors or other methods in future studies.^34^,^35^

Finally, the findings of US during CPR, and hence the decision on resuscitation measures and impact on patient outcomes, could not be easily clarified. Theoretically, this is the most valuable part that US may play during CPR. However, the number of meaningful positive US findings that led to critical therapeutic interventions was small.

## CONCLUSION

Patients receiving ultrasound during resuscitation had comparable chest compression fractions and rates of survival to admission and discharge, and survival to discharge with a favorable neurological outcome when compared to those without US. The individual pause was lengthened related to US. However, patients without US had a shorter resuscitation duration and a better rate of return of spontaneous circulation. The trend toward poorer results in the US group was possibly due to confounding variables or convenience sampling and should be studied in future randomized studies.

## Supplementary Information





## Figures and Tables

**Figure:**
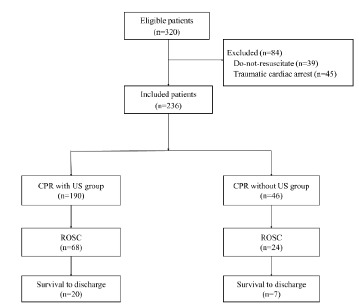
The study diagram. *CPR*, cardiopulmonary resuscitation; *ROSC*, return of spontaneous circulation; *US*, ultrasonography.

**Table 1:** Characteristics of the included patients.

Characteristics	Total (N=236)	US group^e^ (n=190)	Non-US group (n=46)	P-value
Age, years^a^	69 (60.5, 82)	69 (60, 82)	70 (63, 84)	0.64
Male, n (%)	144 (61%)	118 (62%)	26 (57%)	0.49
Medical history, n (%)				
Diabetes mellitus	66 (28%)	57 (30%)	9 (20%)	0.16
Hypertension	127 (54%)	105 (55%)	22 (48%)	0.36
Cardiac disease^b^	90 (38%)	72 (38%)	18 (39%)	0.88
Pulmonary disease^b^	17 (7%)	13 (7%)	4 (8%)	0.66
Renal disease^b^	45 (19%)	34 (18%)	9 (20%)	0.79
Malignancy	42 (18%)	32 (17%)	10 (22%)	0.44
Etiology of arrests, n (%)				
Cardiovascular^c^	114 (48%)	101 (53%)	13 (28%)	<.001
Airway	47 (20%)	36 (19%)	11 (24%)	0.45
Sepsis	20 (8%)	16 (8%)	4 (9%)	0.95
Other^d^	55 (23%)	37 (19%)	18 (39%)	<.001

a Expressed as median (interquartile ranges).

b Cardiac disease included coronary artery disease, heart failure, and arrhythmia; pulmonary disease included bronchial asthma and chronic obstructive pulmonary disease; renal disease included chronic renal insufficiency, and end-stage renal disease receiving dialysis.

c There were 5 patients with myocardial infarction, 5 with dissecting aortic aneurysms, 5 with pericardial effusion, and 2 ruptured abdominal aortic aneurysms in the CPR with US group. Four patients had a myocardial infarction in the CPR without US group.

d There were 10 patients with malignancy, 7 with hyperkalemia, 6 with hypotension, and 14 with unknown causes in the CPR with US group. Seven patients with hypotension, 6 patients with malignancy, 2 with hypoglycemia, 2 with intracranial hemorrhage, and 1 with gastrointestinal bleeding in the CPR without US group.

e Sonographic cardiac activity was detected in 85 patients receiving US. Those with sonographic cardiac activity exhibited a higher chance of the return of spontaneous circulation (64/68 vs 21/122, P<0.0001).

f Comparisons between the two groups.

*CPR*, cardiopulmonary resuscitation; *US*, ultrasonography.

**Table 2:** The cardiac arrest event and resuscitation characteristics.

Characteristics	Total (N=236)	US group (n=190)	Non-US group (n=46)	P-value^d^
Witnessed arrest, n (%)	162 (69%)	129 (68%)	33 (72%)	0.61
Bystander CPR, n (%)	131 (56%)	103 (54%)	28 (61%)	0.41
Pre-hospital CPR duration, minutes^a^	16.0 (5.0, 23.0)	18.0 (5.0, 23.0)	5.0 (4.0, 20.0)	0.18
Initial shockable rhythm, n (%)	24 (10%)	24 (13%)	0	0.06
Defibrillation during BLS and ACLS, n (%)	62 (26%)	55 (29%)	7 (15%)	0.06
In-hospital endotracheal intubation, n (%)^b^	166 (70%)	139 (73%)	27 (59%)	0.06
Epinephrine, mg^a^	8 (6, 12)	8 (6, 12)	7 (3, 12.5)	0.24
Chest compression rate, /minutes^a^	106.5 (101, 112)	108 (101, 112)	105 (101, 108)	0.52
In-hospital resuscitation duration, min^a^	27.7 (11.9, 32.0)	30.3 (13.6, 32.5)	9.7 (7.1, 24.5)	<.001
In-hospital pauses, n^a^,^c^	15 (9, 18.5)	15 (11, 20)	9 (5, 13)	<0.001
In-hospital pause duration, minutes^a^	1.4 (0.9, 2.2)	1.6 (1.0, 2.3)	0.8 (0.4, 1.4)	<0.001
In-hospital chest compression fraction, %^a^	93.5 (90.8, 95.0)	93.0 (91.2, 94.9)	94.3 (89.8, 96.3)	0.29

**Table 3:** Variables for patients receiving ultrasonography during resuscitation.

Variables	Univariate regression Odds ratio (95% CI)	Multiple regression Odds ratio (95% CI)
Age	1.00 (0.98–1.03)	
Gender	1.17 (0.48–2.89)	
Witnessed arrest	0.71 (0.26–1.95)	
Bystander CPR	0.72 (0.29–1.78)	
Pre-hospital CPR duration	1.04 (0.99–1.09)	
Defibrillation during BLS and ACLS	2.06 (0.65–6.52)	
Doses of epinephrine	1.04 (0.95–1.13)	
Cardiac etiology	2.78 (1.06–7.28)^a^	2.02 (0.73–5.58)
In-hospital resuscitation duration	1.08 (1.03–1.13)^b^	1.08 (1.03–1.13)^c^

a P=0.04.

b P=0.02.

c P=0.02.

*CPR*, cardiopulmonary resuscitation; *ED*, emergency department, *CI*, confidence interval.

**Table 4:** Resuscitation outcomes.

Characteristics	Total (N=236)	US group (n=190)	Non-US group (n=46)	P-value^a^
Return of spontaneous circulation, n (%)	92 (39%)	68 (36%)	24 (52%)	0.04
Survival to hospital admission, n (%)	90 (38%)	68 (36%)	22 (48%)	0.13
Survival to hospital discharge, n (%)	27 (11%)	20 (11%)	7 (15%)	0.37
Survival with favorable neurological outcome, n (%)	13 (6%)	9 (5%)	4 (9%)	0.23

a Expressed as median (interquartile ranges).

b In the ultrasound group, 103 patients received one attempt of intubation, 32 received 2 attempts and 4 received 3 attempts. In the non-US group, 26 patients received one attempt, and 1 received 2 attempts.

c Indicated the number of pauses during in-hospital resuscitation.

d Comparisons between the two groups.

*BLS*, Basic Life Support; *ACLS*, Advanced Life Support; *CPR*, cardiopulmonary resuscitation; *US*, ultrasonography.

a Comparison between the two groups.

*US*, ultrasonography.

**Table 5:** Pause duration for pulse checks and the percentage of prolonged pauses.

	US group (190 patients)	Pulse checks alone (n=1,551)	Non-US group (46 patients)
	
	Pulse checks plus US (N=284)		Pulse checks alone (n=234)
Pause duration, seconds^a^	8 (6, 10)^b^	6 (5, 8)^b^	7 (5, 8)
Prolonged pause, n (%)	45 (16%)^c^,^d^	211 (14%)^c^	32 (14%)^d^

a Expressed as median (interquartile ranges).

b P=0.02, compared with the pause for pulse checks with and without US.

c P=0.32.

d P=0.49.

*US*, ultrasonography.
