# Supplementary material for: Impact of Ultrasonography on Chest Compression Fraction and Survival in Patients with Out-of-hospital Cardiac Arrest
Source: West J Emerg Med. 2023 Feb 27;24(2):322–30. doi: 10.5811/westjem.2023.1.58796 (PMC10047717; doi:10.5811/westjem.2023.1.58796)
Supplement: Supplementary file 1 [file wjem-24-322-s001.docx]

| **Supplementary Table 1**. The significant variables for patient outcomes. | | | | | |
| --- | --- | --- | --- | --- | --- |
|  |  | Univariate regression |  |  | Multiple regression |
| Variables |  | Odds ratio (95% CIs) |  |  | Odds ratio (95% CIs) |
| Return of spontaneous circulation |  |  |  |  |  |
| Witness arrest |  | 2.91 (1.24-6.83) |  |  | 3.03 (1.02-8.97) |
| Pre-hospital CPR duration |  | 0.95 (0.91-0.99) |  |  |  |
| In-hospital resuscitation duration |  | 0.90 (0.87-0.94) |  |  | 0.90 (0.87-0.94) |
| Survival to hospital admission |  |  |  |  |  |
| Witness arrest |  | 3.38 (1.40-8.17) |  |  |  |
| Bystander CPR |  | 2.17 (1.03-4.57) |  |  |  |
| Pre-hospital CPR duration |  | 0.95 (0.91-0.99) |  |  |  |
| In-hospital resuscitation duration |  | 0.91 (0.87-0.94) |  |  | 0.90 (0.87-0.94) |
| Survival to hospital discharge |  |  |  |  |  |
| Initial shockable rhythm |  | 6.50 (1.79-23.67) |  |  | 8.37 (2.05-34.16) |
| In-hospital resuscitation duration |  | 0.94 (0.89-0.99) |  |  | 0.93 (0.87-0.98) |

*CPR,* cardiopulmonary resuscitation, *ED,* emergency department, *CI,* confidence interval.

| Supplementary Table 2. The activities during pauses and associated pause durations | | | | |  | |  |
| --- | --- | --- | --- | --- | --- | --- | --- |
|  | 190 patients receiving US | | 46 patients without US | |  |  | |
| Activities during pauses | Times | Pause duration^a^(sec) | Times | Pause duration^a^(sec) |  |  | |
| Pulse checks | 1551 | 6 (5, 8) | 234 | 7 (5, 8) |  |  | |
| Pulse checks with US | 284 | 8 (6, 10) | - | - |  |  | |
| Setting of CPR adjuncts | 273 | 3 (2, 4) | 58 | 3 (2, 4) |  |  | |
| Change of compression provider | 265 | 1 (1, 3) | 49 | 1 (1, 2) |  |  | |
| Ventilation checks | 186 | 7 (6, 8) | 30 | 5 (5, 8) |  |  | |
| Intubation attempts | 179 | 7.5 (4, 11) | 28 | 5.5 (5, 7) |  |  | |
| Bed transfer | 145 | 2 (2, 4) | 29 | 3 (2, 9) |  |  | |
| Defibrillation | 53 | 5 (2, 7) | 5 | 8 |  |  | |
| Setting of ECMO | 17 | 22 (18, 26) | 0 | - |  |  | |
| Pericardiocentesis | 5 | 14 | 0 | - |  |  | |

US=ultrasonography; CPR=cardiopulmonary resuscitation, ECMO=extracorporeal membrane oxygenation.

^a^Median (interquartile range).

Supplementary Table 3. The targets of US scanning during resuscitation.

|  | Pulse checks with US  (n=284) |  |
| --- | --- | --- |
| Scanning targets |  |  |
| Heart | 127  87  70 |  |
| Heart-aorta |  |  |
| Heart-aorta-intraperitoneal free fluid^*^ |  |  |

US=ultrasonography.

^*^Using the focused assessment of sonography for trauma (FAST) to detect free fluid.
