# Supplementary material for: Impact of Ultrasonography on Chest Compression Fraction and Survival in Patients with Out-of-hospital Cardiac Arrest
Source: West J Emerg Med. 2023 Feb 27;24(2):322–30. doi: 10.5811/westjem.2023.1.58796 (PMC10047717; doi:10.5811/westjem.2023.1.58796)
Supplement: Supplementary file 2 [file wjem-24-322-s002.docx]

Basic emergency ultrasound curriculum

| Time frame | Content | | | | |
| --- | --- | --- | --- | --- | --- |
| 13：00~13：10 | Registration | | | | |
| 13：10~14：00 | Introduction | | | | |
| Small group | Cardiac US | Lung US | Abdomen | AAA&DVT  Assessment | Extended FAST |
| 14：00~14：40 | A | B | C | D | E |
| 14：40~15：20 | E | A | B | C | D |
| 15：20~15：30 | BREAK | | | | |
| 15：30~16：10 | D | E | A | B | C |
| 16：10~16：50 | C | D | E | A | B |
| 16：50~17：30 | B | C | D | E | A |
| 17：30~17：40 | Discussion | | | | |

*The ratio of the instructor and trainees was 1:5.

US-CAB Curriculum

| Time frame | Content | | | |
| --- | --- | --- | --- | --- |
| 13：00~13：10 | Registration | | |  |
| 13：20~14：00 | Introduction | | |  |
| Small group | Cardiac US | Airway US | Lung US |  |
| 14 : 00~14: 40 | A | C | B |  |
| 14 : 40~15: 20 | B | A | C |  |
| 15 : 20~16: 00 | C | B | A |  |
| 16 : 00~16: 10 | break | | |  |
| Small group | US-CAB | US-CAB | US-CAB |  |
| 16: 10~17: 00 | A | B | C |  |
| 17: 00~17: 10 | Discussion | | |  |

*The ratio of the instructor and trainees was 1:5.
